# Quantum Obfuscation of Generalized Quantum Power Functions with Coefficient

**DOI:** 10.3390/e25111524

**Published:** 2023-11-07

**Authors:** Yazhuo Jiang, Tao Shang, Yao Tang, Jianwei Liu

**Affiliations:** School of Cyber Science and Technology, Beihang University, Beijing 100083, China; jiangyazhuo@buaa.edu.cn (Y.J.); tang_yao@buaa.edu.cn (Y.T.); liujianwei@buaa.edu.cn (J.L.)

**Keywords:** quantum cryptography, quantum obfuscation, quantum power function, quantum obfuscator, quantum interpreter

## Abstract

Quantum obfuscation is one of the important primitives in quantum cryptography that can be used to enhance the security of various quantum cryptographic schemes. The research on quantum obfuscation focuses mainly on the obfuscatability of quantum functions. As a primary quantum function, the quantum power function has led to the development of quantum obfuscation because it is applicable to construct new obfuscation applications such as quantum encryption schemes. However, the previous definition of quantum power functions is constrained and cannot be beneficial to the further construction of other quantum functions. Thus, it is essential to extend the definition of the basic quantum power function in a more general manner. In this paper, we provide a formal definition of two quantum power functions called generalized quantum power functions with coefficients, each of which is characterized by a leading coefficient and an exponent that corresponds to either a quantum or classical state, indicating the generality. The first is the quantum power function with a leading coefficient, and the second is the quantum *n*-th power function, which are both fundamental components of quantum polynomial functions. In addition, obfuscation schemes for the functions are constructed by quantum teleportation and quantum superdense coding, and demonstrations of their obfuscatability are also provided in this paper. This work establishes the fundamental basis for constructing more quantum functions that can be utilized for quantum obfuscation, therefore contributing to the theory of quantum obfuscation.

## 1. Introduction

In recent years, the development of quantum computing has deeply threatened classical cryptography. More and more quantum algorithms are proven to be effective in breaking classical cryptographic protocols. For instance, Shor’s algorithm [1] reduces the time complexity of solving the difficult problem of large integer factorization to the polynomial level.

Quantum computing not only presents cryptography with new challenges but also with new opportunities. Quantum cryptography is the science of exploiting the superior properties of quantum mechanics to perform cryptographic tasks. In 1984, Bennett and Brassard proposed the BB84 protocol [2], the world’s first quantum key distribution protocol, which utilizes the principle of quantum uncertainty to solve the problems of eavesdropping and man-in-the-middle attacks in key distribution protocols. Subsequently, quantum cryptography has developed a succession of new protocols, including quantum teleportation, quantum secret sharing [3], quantum message authentication [4], and quantum signature [5].

Quantum obfuscation is one of the primitives of quantum cryptography that can be used to enhance the security of quantum cryptographic schemes. The study of quantum obfuscation originated from classical obfuscation. The research on classical obfuscation was originally initiated by Hada [6] and formally elaborated by Barak. Barak et al. [7] analyzed the impossibility of virtual black-box (VBB) obfuscation and proposed a new definition of obfuscation called indistinguishability obfuscation. The definition requires that if two circuits compute the same function, then their obfuscations should be indistinguishable. Lynn et al. [8] discussed point functions and the simple obfuscation of combined point functions and presented the first positive result of obfuscation theory by means of an access control problem based on regular expression. In 2013, Garg et al. [9] gave a general structure for indistinguishable obfuscation and functional encryption applicable to all circuits with polynomial size. Several applications of obfuscation are subsequently demonstrated [10,11,12,13,14], and these works have significantly promoted the study of classical obfuscation.

Quantum obfuscation can enhance the security of quantum cryptographic applications. For instance, quantum point obfuscation can successfully protect private keys and identity information in cryptographic protocols. Furthermore, quantum obfuscation can be used in the construction of various quantum cryptographic protocols, providing greater flexibility. In 2016, Alagic and Fefferman [15] first proposed the definitions of quantum black-box obfuscation and quantum indistinguishable obfuscation. They also constructed general structures for some related cryptography applications, including quantum-secure one-way functions and quantum symmetric encryption. This work has greatly promoted the study of quantum obfuscation. Quantum obfuscation-based cryptographic applications have made some progress in recent years, including the quantum IND-secure quantum symmetric encryption scheme [16], the quantum homomorphic encryption scheme [17], the quantum tokens for digital signatures [18], and the quantum money scheme [19].

Research on the construction of quantum obfuscation is still relatively immature. To further develop the construction methods of quantum obfuscation, it is essential to investigate the obfuscatability of quantum nonlinear functions since they cover a wide range of obfuscatable quantum functions. Pan [20] et al. proposed a universal method for obfuscating quantum parameters in quantum nonlinear functions. They also defined a rough quantum power function to demonstrate universal obfuscation. However, as a basic component of nonlinear functions and composite functions, quantum power functions are not well defined at present. Consequently, it is important to extend the definition of quantum power functions, which we refer to as generalized quantum power functions.

In this work, we first introduce practical primitives for the obfuscation of generalized quantum power functions. We define two specific generalized quantum power functions and illustrate the functionality of each function. Then, we construct obfuscation schemes for generalized quantum power functions and provide demonstrations of their obfuscatability. We hope that our work will be beneficial to the theory of quantum obfuscation.

The main contributions of our work are described as follows:

1. Definition of generalized quantum power functions. We define two different generalized quantum power functions, which we refer to as quantum power functions with leading coefficient and quantum *n*-th power functions, respectively. A quantum power function with a leading coefficient contains two parameters in the form of a quantum state. Quantum *n*-th power function contains both quantum and classical parameters.
2. Construction of quantum obfuscation schemes for generalized quantum power functions. We construct different quantum circuits to obfuscate and interpret each function. We utilize quantum teleportation for multiple qubits to obfuscate two quantum states simultaneously in a quantum power function with a leading coefficient. In addition, we utilize quantum teleportation and quantum superdense coding to obfuscate quantum and classical parameters in the quantum *n*-th power function. More generally, the parameter obfuscation method used in this work can be applied as a general method to obfuscate more quantum functions, therefore contributing to the progression of quantum obfuscation.

## 2. Related Work

### 2.1. Quantum Function Obfuscation

Quantum obfuscation was first introduced by Alagic et al. [15], which is derived from classical obfuscation. The black-box quantum obfuscator was first defined and demonstrated more practicable than the classical black-box obfuscator.

**Definition 1.**  *A black-box quantum obfuscator is a quantum algorithm O and a QPT interpreter δ such that whenever C is an n-qubit quantum circuit, the output of O is an m-qubit quantum state O(C) satisfying*

1. *Polynomial expansion: The output quantum state O(C) with m-qubit remains polynomial scale satisfying*
(1) m=poly(n).
2. *Functional equivalence: For any possible quantum state ρ, there exists*
(2) $$||\delta \left(O\left(C\right)⊗\rho \right)-U_{C}\rho U_{C}^{†}{\left|\right|}_{tr}\leq negl\left(n\right).$$
*The obfuscated program has the same functionality as the original one.*
3. *Virtual black box: For every QPT adversary A there exists a QPT quantum simulator $S^{U_{C}}$ such that*
(3) $$|Pr\left[A\left(O\left(C\right)\right)=1\right]-Pr\left[S^{U_{C}}(|0^{n}\rangle \right)=1]|\leq negl\left(n\right)$$
*Here, poly(n) means any polynomial of n and negl(n) means for all $c\in R_{>0}$, there exists $n_{0}\in Z_{>0}$ such that for all integers $n>n_{0}$, we have $|f\left(n\right)|<1/n^{c}$.*

The end user (and also any adversary *A*) should be in possession of a quantum computer, Most theoretical research on quantum obfuscation focuses on the obfuscation of quantum circuits. The definition of quantum obfuscation for quantum point functions was introduced by Chen et al. [16]. They defined the quantum point function with a general output and constructed an obfuscation scheme for quantum point functions.

**Definition 2.**  *A quantum point function $U_{\alpha ,\beta }$ with a general output is defined as*

(4) $$U_{\alpha ,\beta }:|x,0^{m}\rangle \mapsto |x,P_{\alpha ,\beta }\left(x\right)\rangle ,$$

*where $\alpha \in {\left\{0,1\right\}}^{n},\beta \in {\left\{0,1\right\}}^{m}\setminus 0^{m}$ and $P_{\alpha ,\beta }$ is a classical function expressed as*

(5) $$P_{\alpha ,\beta }\left(x\right)=\left\{\begin{array}{c} \beta ,\;\mathrm{if}\;x=\alpha \\ 0^{m},\;\mathrm{otherwise}\;\; \end{array}\right..$$

Chen et al. demonstrated the obfuscatability of the quantum point function with a general output and proposed a specific quantum obfuscation scheme for the quantum point function. And they also proposed a quantum symmetric encryption scheme based on the quantum point function obfuscation.

However, there are still a few constructions of quantum obfuscation for different quantum functions. The definition of quantum power function was first introduced by Pan et al. [20]. Quantum power function returns |1〉 when the exponent |a〉=|0〉, and returns |x〉 when the exponent |a〉=|1〉.

**Definition 3.**  *A quantum power function $U_{\alpha }$ is defined as*

(6) $$U_{a}:|x,y\rangle \mapsto |x,y⊕P_{a}\left(x\right)\rangle .$$

*Here, a∈{0,1} and $P_{a}$ is a function expressed as*

(7) $$|P_{a}\left(x\right)\rangle =|x^{a}\rangle =\left\{\begin{array}{c} |1\rangle ,\;\mathrm{if}\;|a\rangle =|0\rangle \\ |x\rangle ,\;\mathrm{if}\;|a\rangle =|1\rangle \; \end{array}\right..$$

Pan et al. also proposed a method to construct a quantum obfuscator for quantum power function and proved its correctness. The property of the quantum power function can be extracted into the quantum state |a〉, which they named parameter states. They utilized quantum teleportation to obfuscate this parameter and provided some specific cryptographic quantum applications.

However, we note that the definition of quantum power function is so far a relatively rough one, and there are still many quantum functions to be defined that will benefit the theory of quantum obfuscation.

### 2.2. Quantum Techniques for Obfuscation

Pan et al. [20] first utilized quantum teleportation to construct the obfuscation scheme for quantum power functions. They demonstrated that quantum teleportation is universal to quantum nonlinear functions. The specific explanation is described as follows.

**Theorem 1.**  *Quantum obfuscation based on quantum teleportation is universal to quantum nonlinear functions whose parameter can be represented as a quantum state |a〉, which can be written as*

(8) |x,O(C)〉↦|x,a〉.

*Consider the quantum power function as an example. We can transmit the parameter |a〉 by quantum teleportation to obtain the obfuscated state O(|a〉). The receiver obtains the collapsed state after the measurement and performs corresponding unitary operations to restore the parameter |a〉. The quantum power function is obfuscatable and satisfies all properties of quantum obfuscation through this universal obfuscation method.*

**Figure 1:**
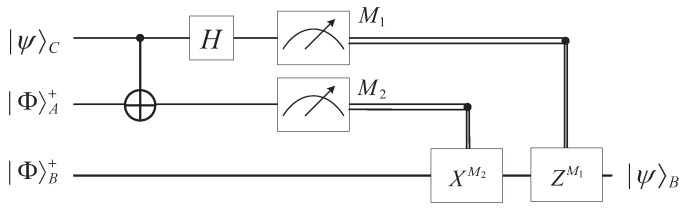
Circuit of quantum teleportation for a single qubit.

Quantum teleportation is used to transfer an unknown quantum state to a distant place by utilizing entangled states. The circuit in Figure 1 gives an accurate description of quantum teleportation.

The relationship between the measurement results, collapsed states, and corresponding unitary operation is listed in Table 1.

**Figure 2:**
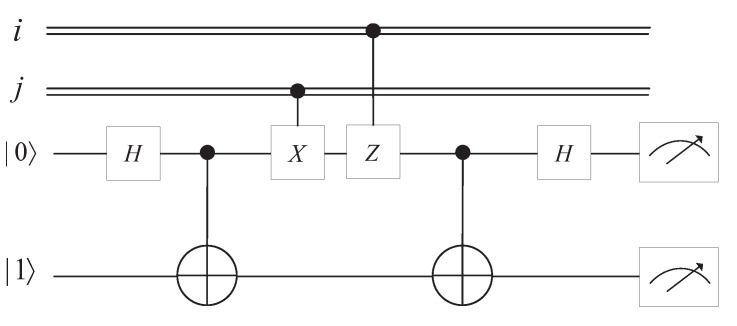
Circuit of quantum superdense coding for 2 classical bits message.

Quantum superdense coding is used to transfer several classical bits of information with a small number of qubits under the assumption of sender and receiver pre-sharing an entangled state. The circuit of quantum superdense coding is illustrated in Figure 2.

The relationship among classical bits, operation of Alice, resultant state, and decoded bits is listed in Table 2.

## 3. Generalized Quantum Power Functions with Coefficient

Quantum power functions are originally defined without coefficients and are restricted to quantum states. We provide a formal definition of two quantum power functions, referred to as generalized quantum power functions with coefficients. Each function is characterized by an exponent that corresponds to either a quantum or classical state, together with a leading coefficient. It is more straightforward to extend quantum power functions with a coefficient into other quantum functions. The first function, which combines the fundamental quantum power function with a leading coefficient in quantum states, is referred to as the quantum power function with a leading coefficient. The second function, which combines both quantum coefficient and classical exponent, is referred to as the quantum *n*-th power function.

### 3.1. Quantum Power Function with Leading Coefficient

According to the theory of quantum computing, classical functions can be mapped to quantum functions by means of quantum circuits. In previous research, quantum power function $\Pi_{a}$ is defined as

(9) $$\Pi_{a}:|x,y\rangle \mapsto |x,y⊕P_{a}\left(x\right)\rangle .$$

Here, a∈{0,1} and $P_{a}$ is a function defined as

(10) $$|P_{a}\left(x\right)\rangle =|x^{a}\rangle =\left\{\begin{array}{c} |1\rangle ,\;\mathrm{if}\;|a\rangle =|0\rangle \\ |x\rangle ,\;\mathrm{if}\;|a\rangle =|1\rangle \; \end{array}\right..$$

A classical polynomial function is a function that can be written as

(11) $$f\left(x\right)=a_{n}x^{n}+\ldots +a_{2}x^{2}+a_{1}x^{1}+a_{0}.$$

Each $a_{i}$ is a coefficient, and each product $a_{i}x^{i}$ is an independent term in the form of a power function with a leading coefficient. To further the development of the quantum polynomial function, we define the quantum power function with a leading coefficient as

(12) $$|y\rangle =|ax^{b}\rangle ,$$

where parameters |a〉 and |b〉 are quantum states, |a〉 is the leading coefficient and |b〉 is the exponent of quantum power function. Equation (13) explains the functionality of quantum power functions with a leading coefficient.

(13) $$|y\rangle =|ax^{b}\rangle =\left\{\begin{array}{c} |0\rangle ,\;\mathrm{if}\;|a\rangle =|0\rangle \\ |1\rangle ,\;\mathrm{if}\;|a\rangle =|1\rangle ,|b\rangle =|0\rangle \\ |x\rangle ,\;\mathrm{if}\;|a\rangle =|1\rangle ,|b\rangle =|1\rangle \end{array}\right.$$

The relationship between quantum coefficient |a〉, quantum exponent |b〉, and the output |y〉 is listed in Table 3.

### 3.2. Quantum n-th Power Function

#### 3.2.1. Bloch Sphere and Qubit Rotation

**Figure 3:**
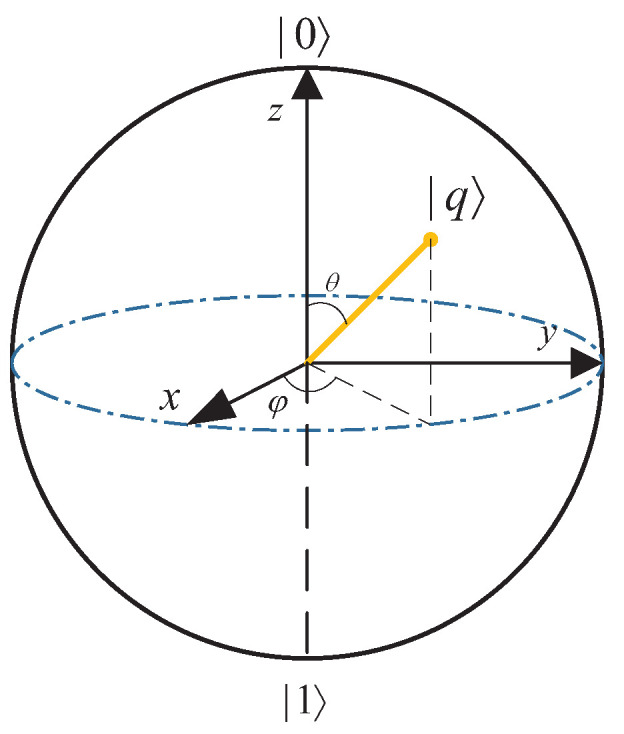
A universal qubit represented by the Bloch sphere.

In quantum computing, the Bloch sphere is a geometrical representation of a universal qubit. The north and south poles of the Bloch sphere correspond to the computational basis vectors |0〉 and |1〉. A universal qubit represented by the Bloch sphere can be written as

(14) $$|q\rangle =\cos\left(\frac{\theta }{2}\right)|0\rangle +\sin\left(\frac{\theta }{2}\right)e^{i\varphi }|1\rangle ,$$

where 0≤θ≤π and 0≤φ≤2π, and it is shown in Figure 3.

Each quantum state vector can also be written in $R^{3}$ as

(15) $$\bar{q}=\left(\sin\theta \cos\varphi ,\sin\theta \sin\varphi ,\cos\theta \right).$$

RX, RY, and RZ gates are rotation operators, indicating the rotation of a qubit through angles α around each axis, and the associated transformation equations for these rotational gates are given by

(16) $$RX\left(\alpha \right)=e^{\frac{-i\alpha X}{2}}=\cos\left(\frac{\alpha }{2}\right)I-i\sin\left(\frac{\alpha }{2}\right)X=\left[\begin{array}{cc} \cos(\frac{\alpha }{2}) & -i\sin(\frac{\alpha }{2})\\ -i\sin(\frac{\alpha }{2}) & \cos(\frac{\alpha }{2}) \end{array}\right]$$

(17) $$RY\left(\alpha \right)=e^{\frac{-i\alpha Y}{2}}=\cos\left(\frac{\alpha }{2}\right)I-i\sin\left(\frac{\alpha }{2}\right)Y=\left[\begin{array}{cc} \cos(\frac{\alpha }{2}) & -\sin(\frac{\alpha }{2})\\ \sin(\frac{\alpha }{2}) & \cos(\frac{\alpha }{2}) \end{array}\right]$$

(18) $$RZ\left(\alpha \right)=e^{\frac{-i\alpha Z}{2}}=\cos\left(\frac{\alpha }{2}\right)I-i\sin\left(\frac{\alpha }{2}\right)Z=\left[\begin{array}{cc} e^{\frac{-i\alpha }{2}} & 0\\ 0 & e^{\frac{i\alpha }{2}} \end{array}\right]=\left[\begin{array}{cc} 1 & 0\\ 0 & e^{i\alpha } \end{array}\right]$$

By combining these operations, qubits can rotate freely on the Bloch sphere.

#### 3.2.2. Functionality of Quantum *n*-th Power Function

According to the classical *n*-th power function, the *n*-th power of a number *x*, when *n* is an integer, is the result of multiplying *x* to itself *n* times (x∗x∗x…∗x). It can be written as $x^{n}$.

Since every qubit can be expressed as (θ,φ), we consider ${|x\rangle }^{1}$ as the final state of rotating |0〉 by angles θ and φ around the Y and Z axes, respectively. According to Equation (19), quantum *n*-th power function can be described as the result of several rotations.

(19) $$|x\rangle^{n}=\cos\left(\frac{n\theta }{2}\right)|0\rangle +\sin\left(\frac{n\theta }{2}\right)e^{ni\varphi }|1\rangle$$

Equation (20) describes the functionality of quantum *n*-th power function. The transformation of |0〉 can be expressed as RZ(nφ)RY(nθ)|0〉, which means the nθ angle is rotated around the y-axis, followed by the nφ angle around the z-axis when |a〉=|1〉. |a〉 is the leading coefficient in quantum *n*-th power functions.

(20) $$|y\rangle =|a\rangle |x\rangle^{n}=\left\{\begin{array}{c} \cos\left(\frac{n\theta }{2}\right)|0\rangle +\sin\left(\frac{n\theta }{2}\right)e^{ni\varphi }|1\rangle ,\;\mathrm{if}\;|a\rangle =|1\rangle \\ |0\rangle ,\;\mathrm{if}\;|a\rangle =|0\rangle \end{array}\right.$$

**Figure 4:**
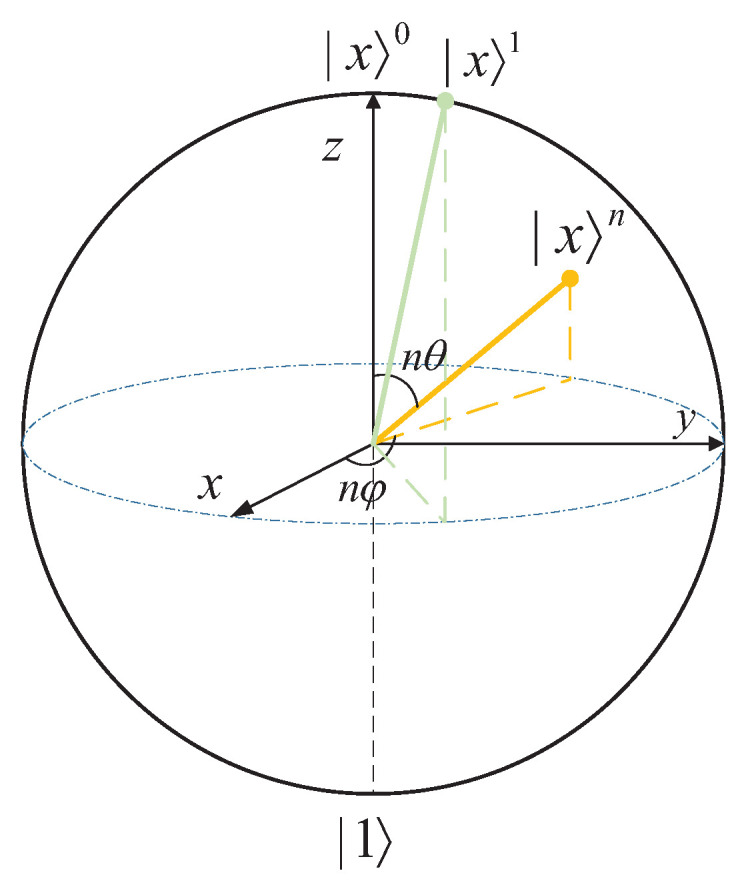
Functionality of quantum n-th power function represented by the Bloch sphere.

The functionality of quantum *n*-th power functions can be visualized in Figure 4.

## 4. Quantum Obfuscation Schemes for Generalized Quantum Power Functions

We propose schemes for obfuscating generalized quantum power functions by quantum teleportation and enhanced quantum superdense coding. Quantum teleportation is used to obfuscate quantum parameters, while enhanced quantum superdense coding is used to obfuscate classical parameters. We construct specific quantum obfuscators to obfuscate each quantum generalized power function and quantum interpreters to restore their original functionality.

### 4.1. Obfuscation Scheme for Quantum Power Function with Leading Coefficient

#### 4.1.1. Construction of Quantum Obfuscator

**Figure 5:**
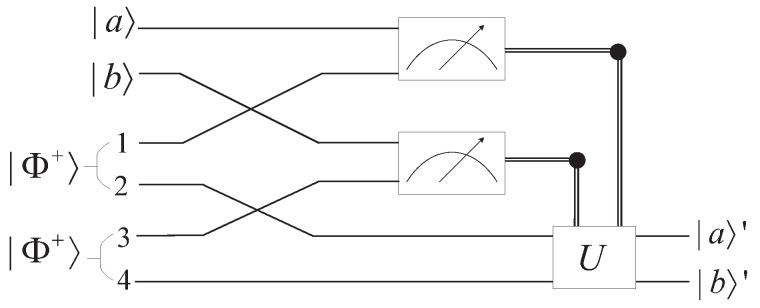
Circuit of quantum teleportation for multiple qubits.

Quantum power function with leading coefficient has the form $|y\rangle ={|a\rangle |x\rangle }^{|b\rangle }$, where |a〉 and |b〉 are both quantum state. To obfuscate two quantum states, we construct the obfuscator by quantum teleportation for multiple qubits [21]. We can teleport the product states of two arbitrary particles independently. Consider the direct product of two quantum states is |Φ〉=|a〉⊗|b〉. The quantum circuit receives two particles as input, while |a〉=α|0〉+β|1〉 and |b〉=γ|0〉+δ|1〉. Particles |a〉,|b〉,1,3 belong to the sender Alice, and particles 2,4 belong to the receiver Bob. The circuit is shown in Figure 5.

Alice measures particles (|a〉,1) and (|b〉,3) by Bell basis. The measurement result of Alice and the unitary operations corresponding to the result are shown in Table 4.

Thus, we can construct an obfuscator for quantum power functions with the leading coefficient by obfuscating the leading coefficient and the exponent. The obfuscation scheme is described as follows.

(1) Alice Input the leading coefficient |a〉, the exponent |b〉 into the obfuscator.
(2) Alice measures the particles (|a〉,1) and (|b〉,3) by Bell basis and obtains the measurement result.
(3) Alice sends the measurement result to Bob through a classical channel.
(4) Bob performs corresponding unitary operations to restore the initial leading coefficient and the exponent of the function.

#### 4.1.2. Construction of Quantum Interpreter

The original quantum circuits, which realize the functionality of quantum power functions with leading coefficients, are obfuscated into qubits through the obfuscator. Consequently, we should design a specific interpreter to explain the obfuscated quantum states and restore the original functionality.

The interpreter is intended to restore the functionality of quantum power functions with a leading coefficient. We can derive a logical expression among the output |y〉, the leading coefficient |a〉, the exponent |b〉 and the input variable |x〉 in Equation (21).

(21) |y〉=(|a〉∧|¬b〉)∨(|a〉∧|¬b⊕x〉)

**Figure 6:**
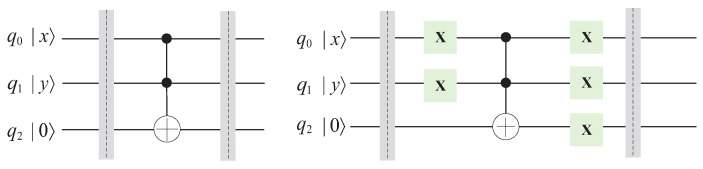
Circuits of quantum “AND” and quantum “OR” gates.

There are no corresponding quantum “AND” and quantum “OR” gates in quantum circuits, so we utilize Toffoli gates to implement quantum “AND” and quantum “OR” gates instead. The circuits are shown in Figure 6.

**Figure 7:**
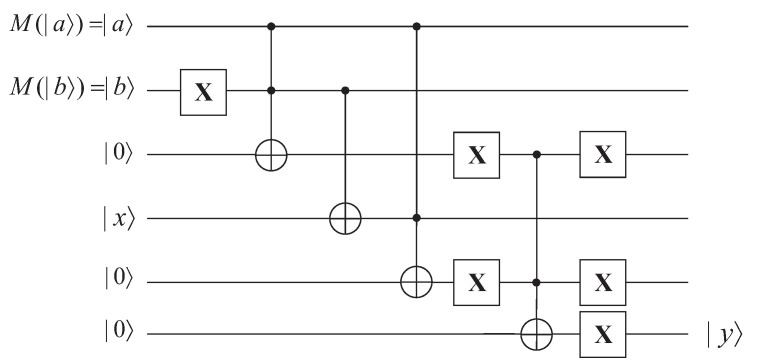
Interpreter of quantum power functions with leading coefficient.

Consequently, we can construct the interpreter by means of corresponding quantum gates to realize the functionality of quantum power functions with the leading coefficient according to the logical expression. Figure 7 shows the quantum circuit of the interpreter.

Initially, receiver Bob performs quantum measurement on the obfuscated results to restore the leading coefficient |a〉 and the exponent |b〉. Then, Bob puts the restored leading coefficient |a〉, the restored exponent |b〉, the base |x〉, and three auxiliary qubits |0〉 into the circuit. The detail of the interpreter is described as follows.

(1) Perform an X gate on the restored exponent |b〉, then we can obtain |¬b〉.
(2) Perform a Toffoli gate on |a〉, |b〉, and the first auxiliary qubit |0〉, in which |a〉 and |b〉 are control qubits and |0〉 is the target qubit. After this step, we obtain |a〉∧|¬b〉.
(3) Perform a CNOT gate on |b〉 and |x〉, in which |b〉 is the control qubit and |x〉 is the target qubit. After this step, we obtain ¬|b〉⊕|x〉.
(4) Perform a Toffoli gate on |a〉,|x〉 and the second auxiliary qubit |0〉, in which |a〉 and |x〉 are the control qubits and |0〉 is the target qubit.
(5) Perform a quantum “OR” gate on |a〉∧|¬b〉 and |a〉∧|¬b⊕x〉. Then we can obtain (|a〉∧|¬b〉)∨(|a〉∧|¬b⊕x〉) which satisfies the functionality of quantum power functions with leading coefficient.

**Theorem 2.**  *Quantum power functions with leading coefficients are obfuscatable by quantum teleportation for multiple qubits. It satisfies all properties of quantum obfuscation.*

**Proof.**  To demonstrate the obfuscatability of quantum power functions with the leading coefficient, we prove the properties of polynomial expansion, functional equivalence, and virtual black-box property as follows.

Assume that quantum power functions with the leading coefficient are denoted by $|y\rangle ={|a\rangle |x\rangle }^{|b\rangle }$, the parameters extracted from it are denoted by $C_{q}(|a\rangle ⊗\left|b\rangle \right)$, which is another representation of the functionality. These parameters are transformed into the obfuscated states $O(C_{q}(|a\rangle ⊗|b\rangle ))$ through the obfuscator.

Suppose the original circuit *C* is *n*-qubit, the obfuscated leading coefficient O(|a〉) is $m_{1}$-qubit and the obfuscated power O(|b〉) is $m_{2}$-qubit. Since all quantum gates contained in the obfuscator operate linearly, the size of $m_{1}$ and $m_{2}$ are certainly of polynomial size. Consequently, the total size of the output is $m_{1}+m_{2}+n$, which is also of polynomial size, satisfying the property of polynomial expansion.

The quantum power functions with the leading coefficient must satisfy the preservation of functionality by the interpreter. In our construction, the two obfuscated parameters can be restored by performing the corresponding unitary operations, which satisfies the property of functional equivalence.

Since the oracle is truly random to any adversary, the obfuscation O(C) leaks no information on the parameters. Thus, it satisfies the virtual black-box property.

Therefore, quantum power functions with the leading coefficient are obfuscatable. □

### 4.2. Obfuscation Scheme for Quantum *n*-th Power Functions

#### 4.2.1. Construction of Quantum Obfuscator

The parameters that need to be obfuscated for quantum *n*-th power functions include both classical and quantum states. Therefore, we utilize quantum teleportation, and enhanced quantum superdense coding [22] to obfuscate the quantum and classical states simultaneously.

**Figure 8:**
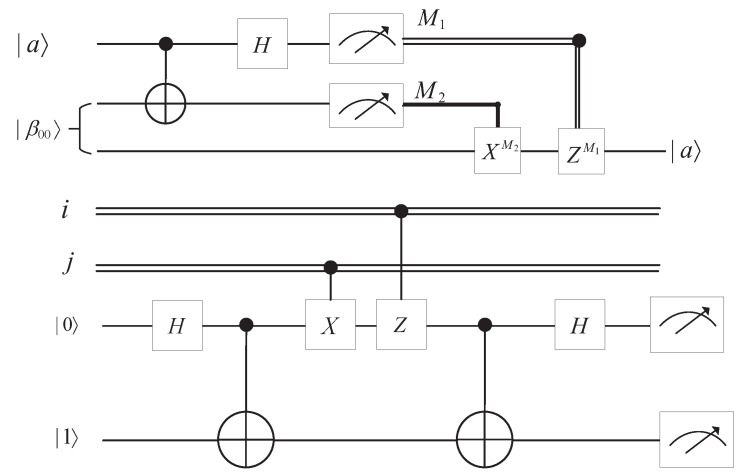
Construction of the obfuscator for quantum *n*-th power functions.

Equation (20) explains the functionality of quantum *n*-th power functions, and Figure 8 shows the obfuscator for quantum *n*-th power functions, assuming the exponent *n* of the function contains two classical bits denoted by ij.

This quantum circuit can be divided into two parts, used to obfuscate quantum parameters and classical parameters, respectively. To obfuscate the leading coefficient |a〉, we use quantum teleportation to obfuscate the qubit |a〉. Depending on different measurements, we perform corresponding unitary operations to restore the initial state according to Table 5.

The classical exponent *n* of quantum *n*-th power function is obfuscated by enhanced quantum superdense coding. First, Alice and Bob generate a pair of Bell states $|\Phi^{+}\rangle_{AB}=\frac{1}{\sqrt{2}}(|00\rangle +\left|11\rangle \right)$ by means of the Hadamard and CNOT gates, each of which holds a qubit. To obfuscate the two-bit parameter *n*, Alice performs the corresponding operation listed in Table 6.

If the exponent of the quantum *n*-th power function is 11, then Alice performs $Z^{1}X^{1}$ to obtain a specific entangled state. Equation (22) demonstrates the specific transformation of the entangled state $|\Phi^{+}\rangle_{AB}$.

(22) $$|\Phi^{+}\rangle_{AB}\overset{X}{\to }\;\frac{1}{\sqrt{2}}(|10\rangle +\left|01\rangle \right)\overset{Z}{\to }\;\frac{1}{\sqrt{2}}(|01\rangle -|10\rangle )=|\Psi^{-}\rangle_{AB}$$

Then, Alice sends the transformed qubit to Bob over a quantum channel, and Bob obtains the whole entangled state. Bob performs the CNOT gate on the entangled state, where the received qubit is the control bit, and the qubit he originally owned is the target bit, then performs the Hadamard gate to the received qubit. The last step is to perform the measurement operation under the computational basis, which restores the exponent *b* of the quantum *n*-th power function.

**Figure 9:**
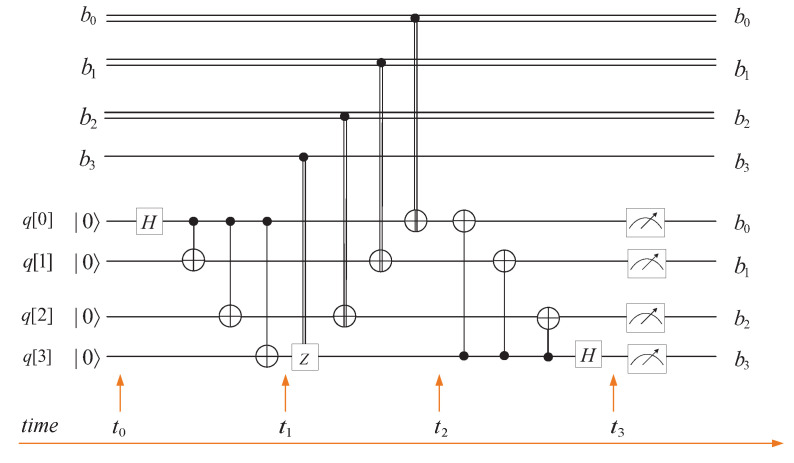
Quantum circuit of the enhanced superdense coding for 4 classical bits.

If the exponent *n* contains multiple bits, it can be obfuscated by means of enhanced superdense coding. This is implemented by constructing the GHZ state, and the circuit for transmitting 4 bits is shown in Figure 9.

**Figure 10:**
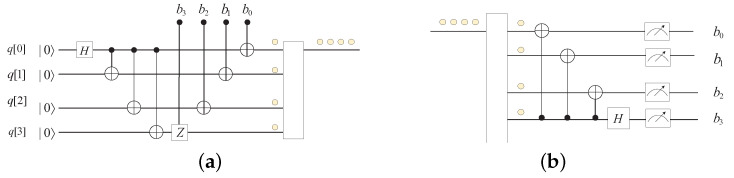
Construction of the obfuscator and the interpreter for 4 classical bits: (**a**) Quantum circuit for obfuscating classical parameters. (**b**) Quantum circuit for restoring classical parameters.

By means of this kind of structure, the number of classical bits to be teleported cannot be restricted anymore. This makes it possible to obfuscate any classical parameters. We split the quantum circuit into two parts to construct the obfuscator and interpreter for classical parameters in Figure 10.

#### 4.2.2. Construction of Quantum Interpreter

**Figure 11:**
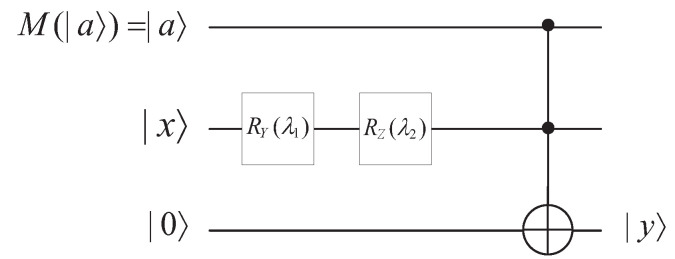
Interpreter of quantum *n*-th power function, where $\lambda_{1}=\left(n-1\right)\theta$ and $\lambda_{2}=\left(n-1\right)\varphi$.

The obfuscator has obfuscated the functionality of the quantum *n*-th power function, so we design an interpreter for the quantum *n*-th power function to restore the parameters and realize the original functionality. The circuit for interpreting the quantum *n*-th power function is shown in Figure 11.

According to the functionality of the quantum *n*-th power function, the parameters to be obfuscated are the hybrid state of quantum and classical states.

The quantum *n*-th power function is characterized by the parameters |a〉 and *n*. When |a〉=|1〉, the rotation on auxiliary qubit |0〉 is RZ(nφ)RY(nθ)|0〉, this occurs by rotation around the y-axis by nθ angles and rotation around the z-axis by nφ angles. If |a〉=|0〉, the output is |0〉.

Consequently, we construct a quantum circuit to implement the functionality. Initially, receiver Bob performs a quantum measurement on the obfuscated results to restore the leading coefficient |a〉 and the exponent *n*. Then, Bob puts the restored leading coefficient |a〉, the base |x〉, and an auxiliary qubit |0〉 into the circuit. The classical exponent, *b*, is reflected in the rotation angle.

The detail of the interpreter is described as follows.

(1) Perform a RY gate on the base |x〉 with the angle $\lambda_{1}$ where $\lambda_{1}=\left(b-1\right)\theta$. Thus, we obtain RY(nθ)|0〉.
(2) Perform a RZ gate on RY(nθ)|0〉 with the angle $\lambda_{2}$ where $\lambda_{2}=\left(n-1\right)\varphi$. Thus, we obtain RZ(nφ)RY(nθ)|0〉.
(3) Perform a Toffoli gate on |a〉, RZ(nφ)RY(nθ)|0〉 and the auxiliary qubit |0〉, where |0〉 is the target qubit. Thus, we obtain the result |y〉=(|a〉∧|x〉)⊕|0〉. When |a〉=|0〉, |y〉=|0〉. When |a〉=|1〉, |y〉=RZ(nφ)RY(nθ)|0〉.

**Theorem 3.**  *Quantum n-th power functions are obfuscatable by quantum teleportation and quantum superdense coding. It satisfies all properties of quantum obfuscation.*

**Proof.**  To prove quantum *n*-th power functions are obfuscatable, we prove its polynomial expansion, functional equivalence, and virtual black-box property as follows.

Assume that quantum *n*-th power functions with the leading coefficient are denoted by $|y\rangle ={|a\rangle |x\rangle }^{n}$, the parameters extracted from it are denoted by $C_{q}(|a\rangle ⊗n)$, which is another representation of the functionality. These parameters are transformed into the obfuscated states $O(C_{q}(|a\rangle ⊗n))$ through the obfuscator.

Suppose the original circuit *C* is *n*-qubit, the obfuscated leading coefficient O(|a〉) is $m_{1}$-qubit and the obfuscated power O(n) is $m_{2}$-qubit. Since all quantum gates contained in the obfuscator operate linearly, the size of $m_{1}$ and $m_{2}$ are certainly of polynomial size. Consequently, the total size of the output is $m_{1}+m_{2}+n$, which is also of polynomial size, satisfying the property of polynomial expansion.

The quantum *n*-th power functions must satisfy the preservation of its original functionality by the interpreter. In our construction, the two obfuscated parameters can be restored by performing the corresponding unitary operation, which satisfies the property of functional equivalence.

Since the oracle is truly random to any adversary, the obfuscation O(C) leaks no information about the parameters. Thus, it satisfies the virtual black-box property.

Therefore, quantum *n*-th power functions are obfuscatable. □

## 5. Discussion

### 5.1. Impossibility and Possibility

Alagic et al. [23] proposed an impossibility result of quantum obfuscation of classical circuits under the assumption that learning-with-errors (LWE) is hard for quantum computers. Their work is based on the work of Barak [7], who demonstrated that a universal obfuscator that obfuscates classical circuits into other classical circuits is impossible. Alagic made a quantum generalization of this obfuscation and extended the impossible result of Barak to quantum settings, proving the impossibility of universal quantum black-box obfuscation.

Following the demonstration by Barak that a universal black-box obfuscation does not exist, two categories of research were established for classical obfuscation. The first objective is to develop and analyze the obfuscation strategy for particular functions; the second is to weaken the definition of black-box obfuscation. Similarly, Alagic et al. demonstrated the impossibility of universal virtual black-box obfuscation in quantum settings, but quantum obfuscation can still be achieved by particular obfuscation methods for specific functions or circuits. In 2023, Bartusek et al. [24] implemented a quantum obfuscation scheme for polynomial-sized pseudo-deterministic quantum circuits. In particular, this scheme can obfuscate the circuit implementing Shor’s algorithm. This work provides a new method to achieve efficient quantum communication. Thus, the feasibility of quantum obfuscation as a tool for quantum software protection has remained wide open, and the obfuscation scheme proposed in our work for a specific generalized quantum power function with coefficient does not conflict with the impossibility result proposed by Alagic.

### 5.2. Obfuscation Circuits in a Noisy Channel

Quantum teleportation and quantum superdense coding are utilized in the construction of the obfuscator and the interpreter. Quantum teleportation requires a noiseless quantum channel to transmit a purely entangled state. However, in practice, the shared entanglement usually becomes degraded because of various decoherence mechanisms, and the noise and decoherence are unavoidable. When applying the proposed obfuscation circuits to realistic channels, we can implement the idealized quantum teleportation circuit and quantum superdense coding circuit through correspondingly improved protocols to enhance the fidelity of each protocol and overcome the effects of severe noise and loss.

There are several methods to overcome the impact of severe noise and loss. Zhao et al. [25] utilized a noiseless amplifier as a pre-device for quantum teleportation and achieved a high fidelity of 92% for teleporting coherent states, which also presented a new way of applying teleportation to purify quantum systems from thermal noise. Islam et al. [26] demonstrated that the decoherence noise can enhance the quantum correlation between two qubits and restore the entanglement lost in the environment, therefore improving the fidelity of quantum teleportation and the capacity of quantum superdense coding and contributing to efficient quantum communication.

## 6. Conclusions

In this paper, we have defined generalized quantum power functions with coefficients and applied quantum mechanics to construct circuits for quantum obfuscators and interpreters. Quantum and classical parameters are obfuscated by quantum teleportation for multiple qubits and enhanced quantum superdense coding, respectively. Also, we have constructed quantum interpreters to restore the functionality of generalized quantum power functions with coefficients.

In the construction of the obfuscation scheme for quantum power functions with leading coefficient, we utilized quantum teleportation for multiple qubits to obfuscate quantum parameters, summarized the logical expression, and utilized the Toffoli gate to realize the quantum “OR” gate and the quantum “AND” gate to construct the interpreter. In the construction of the obfuscation scheme for the quantum *n*-th power function, we utilized quantum teleportation and quantum-enhanced superdense coding to obfuscate both quantum and classical parameters, and we utilized rotational gates for constructing the interpreters to restore the functionality of quantum *n*-th power function. Furthermore, the obfuscatability of generalized quantum power functions with coefficients is demonstrated in this paper.

Other types of quantum functions, such as quantum polynomial functions and quantum composite functions, have not yet been defined, and corresponding obfuscation schemes have not yet been developed either. Also, it remains unexplored how to use the quantum circuit to restore the functionality of an obfuscated quantum function and how to build a simple and efficient quantum interpreter.

## Figures and Tables

**Table 1:** Relationship among measurement results collapsed states, and unitary operations.

Measurement Result	Collapsed State	Unitary Operation
|00〉	α|0〉+β|1〉	$Z^{0}X^{0}$
|01〉	α|1〉+β|0〉	$Z^{0}X^{1}$
|10〉	α|0〉−β|1〉	$Z^{1}X^{0}$
|11〉	α|1〉−β|0〉	$Z^{1}X^{1}$

**Table 2:** Relationship among classical bits, Alice’s operation, resultant state, decoded bits.

Classical Bits	Operation of Alice	Resultant State	Decoded Bits
00	$Z^{0}X^{0}$	$|\Phi^{+}\rangle_{AB}$	00
01	$Z^{0}X^{1}$	$|\Psi^{+}\rangle_{AB}$	01
10	$Z^{1}X^{0}$	$|\Phi^{-}\rangle_{AB}$	10
11	$Z^{1}X^{1}$	$|\Psi^{-}\rangle_{AB}$	11

**Table 3:** Relationship between the parameters and the output.

|ab〉	|y〉
|00〉	|0〉
|01〉	|0〉
|10〉	|1〉
|11〉	|x〉

**Table 4:** Unitary operations corresponding to the measurement result of Alice.

Measurement Result	Unitary Operation	Measurement Result	Unitary Operation
$|\Phi^{+}\rangle_{A1}{|\Phi^{+}\rangle }_{B3}$	$Z^{0}X^{0}⊗Z^{0}X^{0}$	$|\Psi^{+}\rangle_{A1}{|\Phi^{+}\rangle }_{B3}$	$Z^{0}X^{1}⊗Z^{0}X^{0}$
$|\Phi^{+}\rangle_{A1}{|\Phi^{-}\rangle }_{B3}$	$Z^{0}X^{0}⊗Z^{1}X^{0}$	$|\Psi^{+}\rangle_{A1}{|\Phi^{-}\rangle }_{B3}$	$Z^{0}X^{1}⊗Z^{1}X^{0}$
$|\Phi^{-}\rangle_{A1}{|\Phi^{+}\rangle }_{B3}$	$Z^{1}X^{0}⊗Z^{0}X^{0}$	$|\Psi^{-}\rangle_{A1}{|\Phi^{+}\rangle }_{B3}$	$Z^{1}X^{1}⊗Z^{0}X^{0}$
$|\Phi^{-}\rangle_{A1}{|\Phi^{-}\rangle }_{B3}$	$Z^{1}X^{0}⊗Z^{1}X^{0}$	$|\Psi^{-}\rangle_{A1}{|\Phi^{-}\rangle }_{B3}$	$Z^{1}X^{1}⊗Z^{0}X^{0}$
$|\Phi^{+}\rangle_{A1}{|\Psi^{+}\rangle }_{B3}$	$Z^{0}X^{0}⊗Z^{0}X^{1}$	$|\Psi^{+}\rangle_{A1}{|\Psi^{+}\rangle }_{B3}$	$Z^{0}X^{1}⊗Z^{0}X^{1}$
$|\Phi^{+}\rangle_{A1}{|\Psi^{-}\rangle }_{B3}$	$Z^{0}X^{0}⊗Z^{1}X^{1}$	$|\Psi^{+}\rangle_{A1}{|\Psi^{-}\rangle }_{B3}$	$Z^{0}X^{1}⊗Z^{1}X^{1}$
$|\Phi^{-}\rangle_{A1}{|\Psi^{+}\rangle }_{B3}$	$Z^{1}X^{0}⊗Z^{0}X^{1}$	$|\Psi^{-}\rangle_{A1}{|\Psi^{+}\rangle }_{B3}$	$Z^{1}X^{1}⊗Z^{0}X^{1}$
$|\Phi^{-}\rangle_{A1}{|\Psi^{-}\rangle }_{B3}$	$Z^{1}X^{0}⊗Z^{1}X^{1}$	$|\Psi^{-}\rangle_{A1}{|\Psi^{-}\rangle }_{B3}$	$Z^{1}X^{1}⊗Z^{1}X^{1}$

**Table 5:** Unitary operation for specific measurement.

Measurement	Collapsed State	Unitary Operation
|00〉	α|0〉+β|1〉	$Z^{0}X^{0}$
|01〉	α|1〉+β|0〉	$Z^{0}X^{1}$
|10〉	α|0〉−β|1〉	$Z^{1}X^{0}$
|11〉	α|1〉−β|0〉	$Z^{1}X^{1}$

**Table 6:** Obfuscation for two-bit exponent.

Classical Exponent	Operation	States after Operation
00	$Z^{0}X^{0}$	$|\Phi^{+}\rangle_{AB}$
01	$Z^{0}X^{1}$	$|\Psi^{+}\rangle_{AB}$
10	$Z^{1}X^{0}$	$|\Phi^{-}\rangle_{AB}$
11	$Z^{1}X^{1}$	$|\Psi^{-}\rangle_{AB}$

## Data Availability

The original contributions presented in the study are included in the article. Further inquiries can be directed to the corresponding author.
